# A Transparent Ultrasound Array for Real-Time Optical, Ultrasound, and Photoacoustic Imaging

**DOI:** 10.34133/2022/9871098

**Published:** 2022-06-08

**Authors:** Haoyang Chen, Sumit Agrawal, Mohamed Osman, Josiah Minotto, Shubham Mirg, Jinyun Liu, Ajay Dangi, Quyen Tran, Thomas Jackson, Sri-Rajasekhar Kothapalli

**Affiliations:** ^1^Department of Biomedical Engineering, The Pennsylvania State University, University Park, PA 16802, USA; ^2^School of Electrical Engineering and Computer Science, The Pennsylvania State University, University Park, PA 16802, USA; ^3^Penn State Cancer Institute, The Pennsylvania State University, Hershey, PA 17033, USA; ^4^Graduate Program in Acoustics, The Pennsylvania State University, University Park, PA 16802, USA

## Abstract

*Objective and Impact Statement.* Simultaneous imaging of ultrasound and optical contrasts can help map structural, functional, and molecular biomarkers inside living subjects with high spatial resolution. There is a need to develop a platform to facilitate this multimodal imaging capability to improve diagnostic sensitivity and specificity. *Introduction*. Currently, combining ultrasound, photoacoustic, and optical imaging modalities is challenging because conventional ultrasound transducer arrays are optically opaque. As a result, complex geometries are used to coalign both optical and ultrasound waves in the same field of view. *Methods*. One elegant solution is to make the ultrasound transducer transparent to light. Here, we demonstrate a novel transparent ultrasound transducer (TUT) linear array fabricated using a transparent lithium niobate piezoelectric material for real-time multimodal imaging. *Results*. The TUT-array consists of 64 elements and centered at ~6 MHz frequency. We demonstrate a quad-mode ultrasound, Doppler ultrasound, photoacoustic, and fluorescence imaging in real-time using the TUT-array directly coupled to the tissue mimicking phantoms. *Conclusion*. The TUT-array successfully showed a multimodal imaging capability and has potential applications in diagnosing cancer, neurological, and vascular diseases, including image-guided endoscopy and wearable imaging.

## 1. Introduction

Ultrasound and optical imaging modalities are nonionizing, portable, affordable and can be realized in various forms, from table top size to miniaturized endoscopes or wearable devices [1, 2]. Ultrasound (US) imaging provides the deep tissue structural information based on differences in acoustic impedance and complementary functional blood flow information through Doppler ultrasound [3]. Pure optical imaging methods such as fluorescence imaging enable biochemical information of targeted cells and tissue (e.g., autofluorescence from metabolic cofactors NAD/NADH: nicotinamide adenine dinucleotide) and therefore allow high diagnostic sensitivity and specificity [4–7]. Optical imaging provides best spatial resolution (submicrons to a few microns) when probing superficial depths (<1 mm). However, strong scattering of optical photons inside the deep tissue severely limits the spatial resolution of pure optical imaging, which is typically in the range of 1/5^th^ to 1/10^th^ of an imaging depth [8]. Photoacoustic (PA) imaging, as a hybrid imaging modality, maps optical absorption contrast of deep tissue with ultrasonic spatial resolution. For example, hemoglobin absorption-based label-free imaging of vascular anatomy and functional oxygen saturation has been shown to be useful in diagnosing cancer, neurological, and vascular diseases [9–11]. In PA imaging, light undergoes only one way scattering inside the tissue medium that is from the skin surface to the target location. At the target location, such as a blood vessel, light is converted to ultrasound waves by light-absorbing chromophores. Because the generated ultrasound waves are about 100-fold less scattered than the light waves, PA imaging provides higher imaging depth and better spatial resolution (scalable with ultrasound parameters, typically 1/100^th^ of an imaging depth, that is 0.5 mm spatial resolution at 5 cm depth) compared to deep tissue optical imaging [8, 12, 13]. While PA imaging provides rich optical contrast from a wide range of light absorbing particles (e.g., proteins, small molecules, and nanoparticles), it is to be noted that the penetration depth and spatial resolution in PA imaging is still lower than conventional ultrasound imaging. Therefore, a synergistic integration of optical, US, and PA imaging technologies into a single multimodal imaging platform will provide complementary contrasts, penetration depths, and spatial resolutions. They are desired in many biomedical applications to simultaneously image a set of structural, functional, and molecular biomarkers.

Different combinations of optical and ultrasound imaging systems have been reported for different clinical applications. In cancer imaging, Fatakdawala et al. demonstrated *in vivo* imaging of oral cancer in a hamster model using a bench-top combination of fluorescence lifetime (FLI), PA and US imaging techniques [14]. FLI revealed biochemical (NADH) changes on the tissue surface, with a lower fluorescence lifetime for the oral cancer tissue compared to the surrounding tissue. US imaging provided underlying tissue morphology and microstructure, and PA imaging detected high vascularization within the cancerous tissue. Similarly, Tummers et al. performed multimodal US, PA, and fluorescence imaging of a surgical removed pancreatic specimen obtained from a pancreatic ductal carcinoma (PADC) patient [15], who was intravenously administered with a near-infrared (NIR) fluorescent agent, Cetuximab-IRDye800, that binds to epidermal growth factor receptor. In this case, fluorescence imaging provided the surface projection of the targeted Cetuximab-IRDye800 agent, PA imaging showed the depth resolved optical absorption contrast from the IRDye800 and surrounding vasculature, and ultrasound imaging revealed the underlying tissue anatomy. For imaging atherosclerosis, a cardiovascular disease characterized by the accumulation of lipid plaques and several fibrous and cellular constituents, intravascular ultrasound (IVUS) and optical coherence tomography (OCT) technologies are commonly used in the clinics [16–18]. Recently, intravascular PA (IVPA) is also being actively studied for mapping deep tissue atherosclerosis based on high optical absorption contrast of plaque lipids in the NIR-IIb (1.5 *μ*m-1.7 *μ*m) optical window [19–23]. Similarly, neuroscience studies also require high-resolution multiparametric hemodynamic information (cerebral blood flow, blood volume, and oxygen saturation) obtained from optical and photoacoustic imaging for mapping resting state brain connectivity [24–26], studying neuromodulation [27], neurovascular coupling [28–30], and neurodiseases [31–33]. For this purpose, recently functional ultrasound (fUS) imaging, which provides high resolution images of microvascular blood flow, has been integrated with hemoglobin absorption-based PA vascular imaging [34].

**Figure 1:**
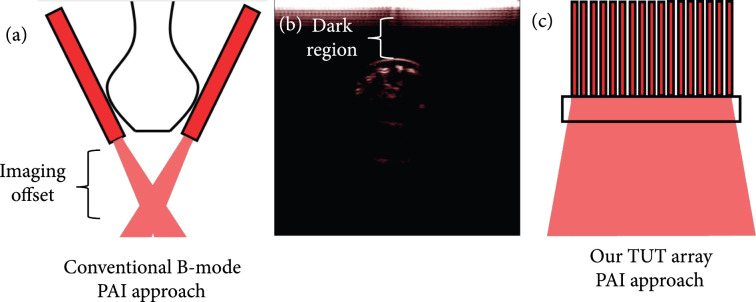
Photoacoustic tomography setup that employs a linear array. (a) Light needs to be attached from the sides, creating imaging offset. (b) A photoacoustic image of the finger shows the dark region that lacks the illumination. Acquired by Acoustic X. However, (c) a transparent linear array allows the light to be coupled from the backside and removes the imaging offset.

However, the current experimental setups integrating fluorescence and US and PA technologies are limited to raster scanning the imaging device over the tissue sample, one imaging mode at a time [14]. Since real-time US imaging (e.g., IVUS and fUS) is performed using ultrasound transducer array, the most viable approach for real-time multimodal imaging is to integrate fluorescence (or other optical technologies) and PA imaging to the US imaging array-based platform. However, optical opacity of conventional ultrasound transducers hinders coaxial and compact integration of the ultrasound transducer array with optical illumination and detection fibers. For example, real-time B-mode US and PA (USPA) imaging devices are developed by simply assembling optical fiber bundles around a conventional ultrasound transducer probe (Figure 1(a)). Due to the physical separation between the two optical fiber bundles, optical illumination is not available below the surface of the ultrasound transducer up to 1 -2 cm depth (see Figures 1(a) and 1(b)) [35, 36]. To partially offset this problem and achieve coaligned optical and ultrasound fields on the tissue surface, USPA devices are operated with long working distances (>1 cm), visible as dark region (Figure 1(b)), using water or ultrasound gel as the coupling medium between the tissue and the probe surface [37, 38]. This limits miniaturization of the multimodal imaging devices and longitudinal *in vivo* imaging capabilities, introduces artifacts, and increases ultrasound attenuation as well as ultrasound scattering if any bubbles are formed in the coupling medium. The requirement for long working distance also limits the imaging speed because of redundant data corresponding to nonilluminated region is also captured and processed both during US and PA data acquisition system. For example, the additional working distance required for real-time PA imaging will preclude its integration with power Doppler ultrasound- (PDUS-) based microvasculature imaging that needs high-frame rate (>10,000 frames per second) plane wave ultrasound imaging [3, 39].

The above challenges can be overcome by employing transparent ultrasound transducers (TUT) that allow light delivery through the transducer, as shown in Figure 1(c). By doing so, the ultrasound transducer becomes a part of the optical system, instead of an obstruction to the optics. This will not only significantly reduce the beam engineering challenges but will also lead to the development of a more compact, portable, wearable, and versatile multimodal systems. For this purpose, both conventional piezoelectric materials [40–43] and capacitive micromachined ultrasound transducers (CMUTs) [44–46] have been studied for developing TUTs. Ilkhechi et al. reported transparent CMUT array for ultrasound imaging of a small size tissue phantom [45] and photoacoustic [46] imaging of pencil leads submerged in oil tank. Transparent CMUTs have not yet been demonstrated for deep tissue US and real-time dual-modality USPA imaging capabilities. Although CMUTs have unique advantages such as wide bandwidth and ease of fabrication in 1D and 2D arrays forms with different shapes, sizes, and frequencies, they require complex clean room fabrication processes, large bias voltages, and custom-developed integrated circuits for operation, leading to their incompatibility with current clinical ultrasound systems [10, 47–49]. All-optical photoacoustic detectors such as transparent optical ring resonators [50] and Fabry–Pérot etalons [51] have the ability to transmit light through them and into the tissue. However, these systems require additional laser and optical detectors for detecting generated photoacoustic waves, and as such not compatible with commercial ultrasound machines [52]. Moreover, these detectors cannot be used for ultrasound excitation/imaging required for dual-modality USPA imaging applications. While prior studies demonstrated the potential of transparent lithium niobate- (LN-) based single element TUTs for high sensitivity PA imaging [40–43], TUT-arrays are required for real-time multimodal imaging.

To address above-mentioned limitations, in this work, we introduced the one-dimensional (1D) linear TUT-array using a transparent LN piezoelectric material and demonstrated its feasibility for a real-time multimodal deep tissue imaging. To the best of our knowledge, this is the first TUT-array which uses a transparent bulk piezoelectric material. We characterized the TUT-array using electrical and acoustic methods. The TUT-array enabled coalignment of acoustic and light pathways with minimal acoustic coupling. Imaging of tissue mimicking phantoms validated a quad-mode US, PA, Doppler ultrasound, and fluorescence imaging capabilities of the TUT-array for providing respective structural, functional, and molecular information of the tissue without introducing any shadow regions. In the future, TUT-arrays can have broad biomedical applications such as compact multimodal endoscopy or wearable imaging applications and also for photo-mediated ultrasound therapy for deep vein thrombosis or wound healing.

## 2. Results

### 2.1. TUT-Array Design and Fabrication

**Figure 2:**
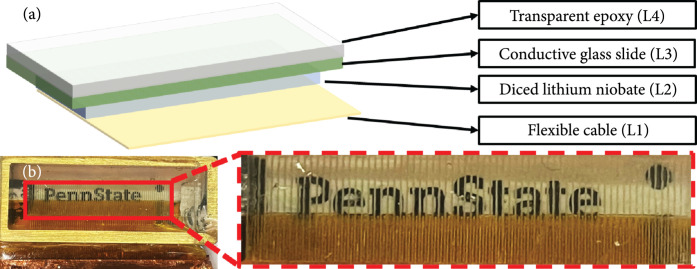
(a) Schematic of the transparent ultrasound transducer array (TUT-array) using double-sided ITO-coated transparent lithium niobate piezoelectric material. The matching layer of Parylene is not shown. (b) A photograph of the fabricated TUT linear array on top of the “Penn State” logo.

The schematic of the proposed TUT-array is shown in Figure 2(a). The Krimboltz, Leedom, and Mattaei (KLM-) model-based simulation software (PiezoCAD, Sonic Concepts, Woodinville, WA, USA) [53] was used to study the electrical impedance, pulse-echo response, and corresponding bandwidth of the array element, while MATLAB Ultrasound Toolbox (MUST) was used to simulate the beam profile of the 16-element synthetic aperture of the array for different steering angles [54]. A center frequency of 6.5 MHz was chosen to match commonly used diagnostic ultrasound devices. This can be achieved by a 0.5 mm thick LN piezoelectric material. Double side indium tin oxide- (ITO-) coated LN was selected as the piezoelectric material due to its high optical transmission rate (>80% in the NIR wavelengths) and good electromechanical coupling coefficient (49%). The element width of 0.2 mm was chosen to be less than 0.6× (element thickness) and greater than λ/2 to avoid spurious resonant modes. Here, λ represents the ultrasound wavelength in tissue medium. When designing element pitch, it needs to be within the range from λ/2 to 3λ/2 to avoid grating lobes. Therefore, a pitch of 0.3 mm was chosen for a 6.5 MHz linear array [55] with a total of 64 elements and an element height of 5 mm. 64 elements were created by dicing 400 *μ*m deep inside the 500 *μ*m LN wafer, leaving 100 *μ*m for shorting all elements as the common ground. A 1 mm thick conductive glass slide was bonded to the LN which served as the first backing layer as well as the ground connection. An additional backing layer of transparent epoxy was placed on top of the glass slide to further reduce the acoustic reverberation. To individually address each element, a custom fabricated cable was anisotropic conductive film (ACF) bonded to the edge of the array as shown in Figures 2(a) and 2(b). To improve the ultrasound energy transmission, a quarter wavelength thick matching layer of Parylene-C (not shown in Figure 2(a) schematic) was deposited for acoustic impedance matching and waterproofing. The acoustic properties of the stacking materials used in the TUT-array fabrication are summarized in Table 1 and the design parameters of the array are summarized in Table 2.

**Table 1:** Acoustic properties of the stacking materials in TUT-array fabrication.

Material	Purpose	c(mm/*μ*s)	Z(MRayls)	*α*(dB/cm/MHz)
LiNbO${\;}_{3}$ (L2 in Figure 2(a)) [56]	Active material	7.36	34.5	
Parylene C [56]	Matching layer	2.35	2.585	0.1
Glass (L3 in Figure 2(a)) [57]	First backing	5.8	14.56	0.06
Epotek 301 (L4 in Figure 2(a)) [58]	Second backing	2.64	2.85	1

c: longitudinal speed of sound; Z: acoustic impedance; α: longitudinal acoustic attenuation.

**Table 2:** Design parameters for TUT-array.

Parameters	Values
Center frequency	6.5 MHz
Number of elements	64
Pitch	0.3 mm
Element size T×H×W	0.5 mm×5 mm×0.2 mm
Electrode thickness (ITO)	200 nm
Backing thickness (glass + epoxy)	10 mm
Matching thickness (Parylene)	80 μm

T: thickness; H: height; W: width. ITO: indium tin oxide.

Further, the detailed TUT-array step-wise fabrication is presented in Section 4.1. Figure 2(b) shows the picture of the fabricated TUT linear array on top of a “Penn State” logo. Although discontinuity was observed between the elements due to the dicing kerf, the letters are clearly readable throughout the TUT-array. The zoomed in image in Figure 2(b) shows proper alignment and bonding between flexible cable traces and each LN element.

### 2.2. TUT-Array Characterization

Following the fabrication, various tests to the TUT-array were conducted to determine its electrical and acoustic characteristics and their consistencies across all 64 elements.

#### 2.2.1. Pulse-Echo Measurements

**Figure 3:**
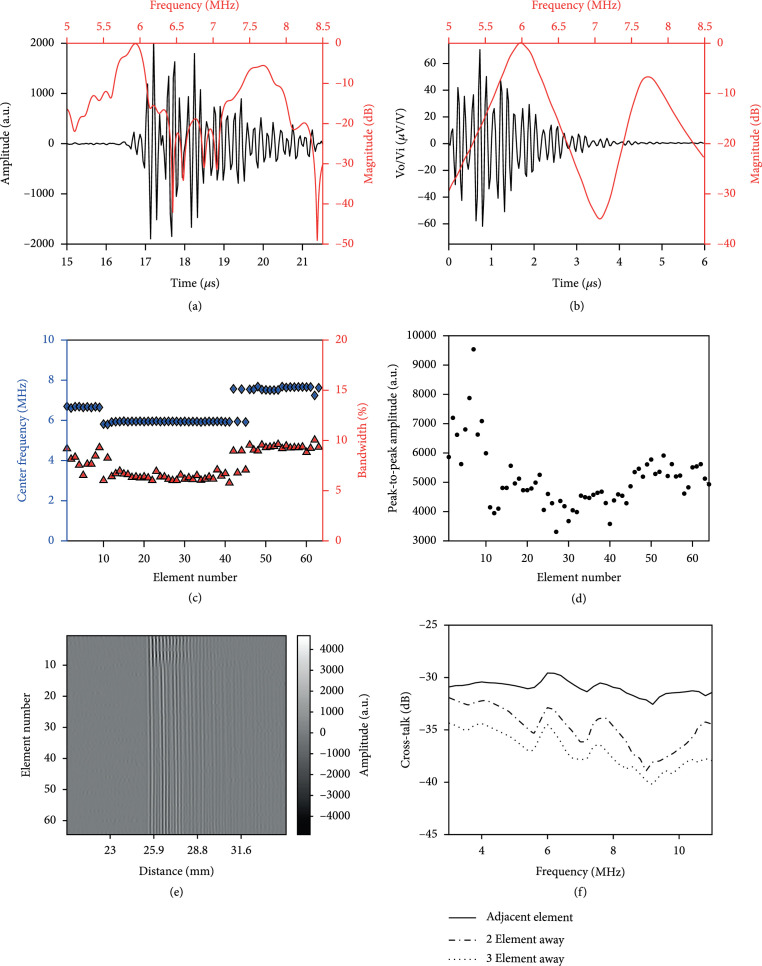
The TUT-array pulse-echo amplitude, frequency, and crosstalk characterization results: (a) experimental and (b) simulated two-way pulse-echo response for center element #32. The black line represents the time domain signal and the red line represents the corresponding frequency response. The pulse-echo characterization was conducted for all 64 elements. (c) The plot of quantified center frequencies and bandwidths for each element. (d) Peak-to-peak pulse-echo amplitude for all 64 elements and (e) the corresponding pulse-echo B-scan lines. (f) The combined electrical and acoustic crosstalk between adjacent elements measured at frequencies in the range of 3 MHz to 11 MHz for element #32.

Pulse-echo characterization was carried out by placing a flat metal target at~26 mm away from the TUT-array, while each element was excited with electrical pulses of 40 volts amplitude from the Vantage 256 (Vantage 256, Verasonics Inc., Kirkland, WA, USA). Figure 3(a) shows a typical pulse-echo result obtained from the center element #32 of the array. Due to the mass loading effect from the attached glass slide, a dual frequency nature was observed similar to previously reported articles [59, 60] and agreed well with the PiezoCAD simulation, as shown in Figure 3(b). The center frequencies of the element were found to be 5.94 MHz and 7.69 MHz, with a -6 dB fractional bandwidths of 6.2% and 7.6%, respectively. These results were similar to characteristics found in the simulation with 5.96 MHz and 7.20 MHz center frequencies with respective fractional bandwidths of 6.58% and 5.34%. To investigate the consistency across all the 64 array elements, the center frequencies and corresponding bandwidths of each element are plotted in Figure 3(c). The plotted center frequency only indicated the more dominant frequency, which exhibited the higher magnitude in frequency response (0 dB after normalization). The two-way pulse-echo peak-to-peak amplitudes for each array element are plotted in Figure 3(d) and corresponding B-scan images from all elements are plotted in Figure 3(e). This data can be categorized into three subgroups: *subgroup 1*: element #1 to #9; *subgroup 2*: element #10 to #41; and *subgroup 3*: element #42 to #64, with center frequencies to be 6.65 MHz, 5.93 MHz, and 7.41 MHz, respectively, and with corresponding averaged bandwidths of 8.1%, 6.45%, and 9.12%. Subgroup 1 showed significantly higher peak-to-peak amplitude than other two groups, while subgroup 2 showed the lowest peak-to-peak amplitude. We hypothesize that these differences were due to the uneven residual bonding epoxy thickness between the LN (Figure 2(a) L2) and the backing conductive glass (Figure 2(a) L3). To further confirm this, we performed PiezoCAD simulations of a TUT transducer element with varying residual epoxy thicknesses and compared with the experimental pulse-echo waveforms from three subgroups. The summary of these comparison results is plotted in Supplementary Figure S1, and it shows that the simulated pulse-echo and frequency responses for epoxy thicknesses 0 *μ*m, 15 *μ*m, and 30 *μ*m closely match with the experimental pulse-echo waveforms of elements number 32 (E32), 55 (E55), and 4 (E4) in the array from subgroups 2, 3, and 1, respectively. For example, the simulated center frequencies were found to be 6.90 MHz, 5.99 MHz, and 7.23 MHz, respectively, for the residual epoxy thicknesses of 30 *μ*m, 0 *μ*m, and 15 *μ*m, which are closely matched with the averaged center frequencies of the subgroups 1, 2 and 3, respectively.

#### 2.2.2. Crosstalk Measurements

Due to the subdicing of the TUT linear array in this work, a higher crosstalk between the elements was expected. To quantify the combined acoustic and electrical crosstalk, the TUT-array was placed in a tank with deionized water against a high frequency acoustic absorber (Aptflex F28, Precision Acoustics, Dorchester, UK). The TUT-array element #32 was fired by a function generator with 10 Vpp, 10-cycle burst, with frequency swept from 3 MHz to 11 MHz. The received voltages at the first, the second, and the third adjacent elements were measured and referenced to the excited voltage to assess the combined electrical and acoustic crosstalk [61]. As shown in Figure 3(f), the measured highest crosstalk was found to be -29.6 dB, -32.9 dB, and -34.49 dB for the first, the second, and the third adjacent elements, respectively, at 6 MHz. These crosstalk values were not significantly higher than<-33 dB crosstalk reported for linear arrays at similar frequency [62], which could possibly be due to the lower sensitivity of the array elements. Therefore, crosstalk at element #4 was measured across the same frequency range and shown in Supplementary Figure S2. Due to the higher sensitivity of subgroup 1 comparing to subgroup 2 (Figure 3(d)), the crosstalk was increased to -26 dB at 3 MHz. Interestingly, no significant fluctuations of crosstalk were observed near the resonance frequency (~6.65 MHz for this subgroup), which may be due to the increased residual epoxy thickness for better acoustic absorption (1 dB/cm/MHz acoustic attenuation from Epotek 301 versus 0.06 dB/cm/MHz acoustic attenuation from glass).

#### 2.2.3. Electrical Impedance Measurements

**Figure 4:**
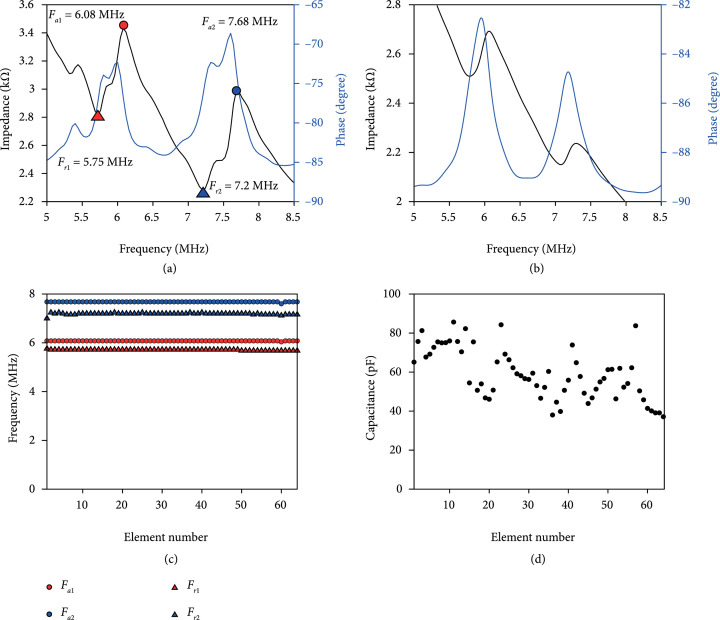
TUT-array electrical impedance characterization results: (a) the experimental and (b) simulated (element #32) single array element input electrical impedance curves. The blue line represents the phase of electrical impedance in degree, and black line represents the electrical impedance in kOhm. (c) The first antiresonance frequency $\left({\text{F}}_{a1}\right)$, the second antiresonance frequency $\left({\text{F}}_{a2}\right)$, the first resonance frequency $\left({\text{F}}_{r1}\right)$, and the second resonance frequency $\left({\text{F}}_{r2}\right)$ for each array element. (d) The measured capacitance in pF for each of 64 array elements.

The electrical impedance measurements were conducted for each element of the TUT-array and the characterization method was described in our previous literature [41]. A calibrated electrical impedance analyzer (Agilent 4990A, Keysight Technologies, Inc., Santa Rosa, CA, USA) was used to determine the phase and electrical impedance for each linear array element. Figure 4(a) shows the measured input impedance magnitude and phase plots for the center element: #32. Due to the dual frequency exhibited in these elements, two pairs of resonance $\left({\text{F}}_{r}\right)$ and antiresonance $\left({\text{F}}_{a}\right)$ frequencies were observed. The first pair resonance $\left({\text{F}}_{r1}\right)$ and antiresonance frequency $\left({\text{F}}_{a1}\right)$ were found to be 5.75 MHz and 6.08 MHz, respectively, and the second pair has resonance $\left({\text{F}}_{r2}\right)$ and antiresonance frequency $\left({\text{F}}_{a2}\right)$ of 7.2 MHz and 7.68 MHz, respectively. The electromechanical coupling coefficient was then calculated to be 0.325 and 0.348 for the two pairs according to the IEEE standard on piezoelectricity [63]. These resonance and antiresonance frequencies agreed well with the PiezoCAD simulation as shown in Figure 4(b), although discrepancies at the impedance values were observed due to the limitations of simulating the system electrical resistance, primarily from the high resistivity of ITO, with PiezoCAD. Then to examine the uniformity in electrical impedance across all elements, two pairs of the F${\;}_{r}$ and F${\;}_{a}$ for each array element are plotted in Figure 4(c). Interestingly, the variations from pulse-echo measurement were not present in the electrical impedance measurement. PiezoCAD simulation of electrical impedance of array elements for the residual epoxy thicknesses of 0 *μ*m, 15 *μ*m, and 30 *μ*m and the corresponding experimental results from the three subgroups were shown in Supplementary Figure S3. The simulation results for the 0 *μ*m and 15 *μ*m thick residual epoxy closely matched in both magnitude and phase impedance curves with typical elements E32 and E55 from subgroups 2 and 3. However, the 30 *μ*m epoxy simulation result exhibits slight differences in F${\;}_{r}$ and F${\;}_{a}$ values in comparison with experimental E4 element from subgroup 1, which can be attributed to nonhomogeneous residual thickness on the element that was not able to be simulated. Furthermore, the same impedance analyzer was used to measure the capacitance from each element, and the results are shown in Figure 4(d). Overall, the capacitance across 64 elements ranged from 40 pF to 80 pF, and the observed variations could be largely due to the uneven bonding thicknesses across the array. The first 9 elements showed higher capacitance than the rest of the elements, which may be contributed by the thicker residual epoxy.

#### 2.2.4. Beam Profile Mapping

**Figure 5:**
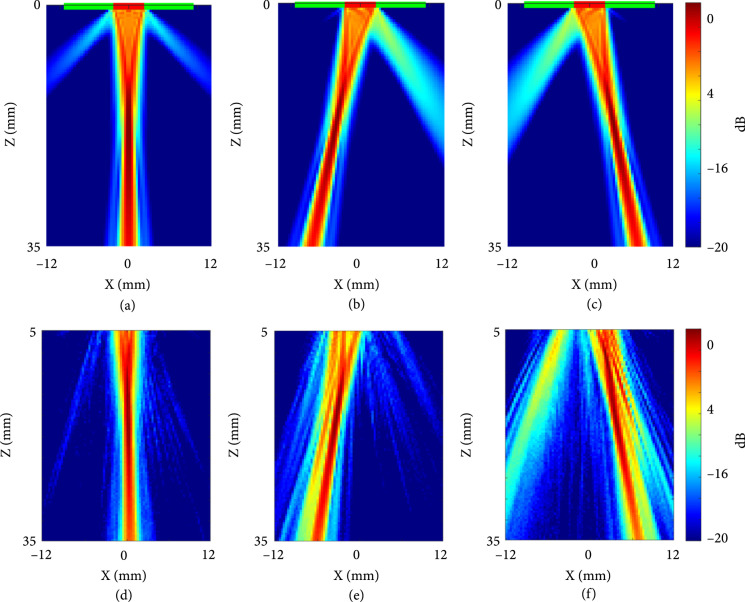
(a) The simulated beam profile at 0 degree, (b) -10 degree, and (c) 10 degree. (d) The hydrophone measured experimental beam profile at 0 degree, (e) -10 degree, and (f) 10 degree. The transmit beam focus was set at z=15 mm in both the simulation and experiment transmission profile.

As the current TUT-array was fabricated using subdicing method with a higher chance of grating lobe artifacts, to generate quality B-mode ultrasound images, we used a focused synthetic aperture beam transmitting strategy with a 16-element effective aperture at 0-degree beam steering and a focus at 15 mm away from the transducer surface. Therefore, in order to evaluate the performance of the proposed linear array in side lobes and grating lobes, the beam profile from 16 elements centered around element #32 and steered at 0, -10, and 10 degrees, was experimentally measured using a scanning hydrophone, and compared with the corresponding simulated beam profiles generated using the MUST software package. The hydrophone measurement procedure is similar to previously reported literature [64] and described in detail in Section 4. The results in Figure 5 show simulated and corresponding experimental beam profiles in the top and bottom rows agreed well overall for three angles: no grating lobes are observed when the beam transmitted at 0 degree (Figures 5(a) and 5(d)), but strong grating lobes were observed when the beam was steered at an angle (Figures 5(b), 5(c), 5(e), and 5(f)). The deteriorated focusing capability shown in experimental results, especially at 10 and -10 degrees, can primarily be due to the subdicing method on the TUT-array.

### 2.3. Quad-Mode Imaging Validation

**Figure 6:**
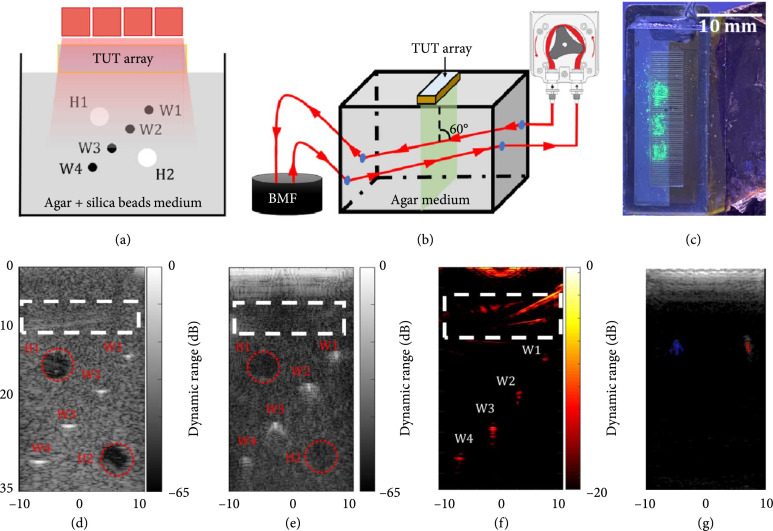
The TUT-array-based multimodal imaging validation. Phantom schematics for (a) ultrasound (US) and photoacoustic (PA) imaging and (b) Doppler ultrasound imaging. BMF: blood-mimicking fluid. Imaging results showing (c) the fluorescence image of the ultraviolet excitable beads patterned as letters “PSU” imaged through the TUT-array. (d) US image from a commercial L7-4 ultrasonic linear array probe and (e) US image from the proposed TUT-array show acoustic impedance contrast from the four micrometal wires (W1-W4) and two hypoechoic targets (H1 and H2 in red dotted circles). (f) Photoacoustic image shows optical absorption contrast from the same four micrometal wires (W1-W4). (g) The coregistered US and color Doppler US image of the phantom in (b) shows the ultrasound speckle contrast in grayscale and the Color Doppler US image in color scale. While the BMF flowing tubes showed the same US contrast, the opposite flow directions of BMF in the two tubes are color coded in blue and red. Panels (d), (e), (f), and (g) have axes units in mm. The white dashed box highlighted in (d), (e), and (f) shows the coupling artifacts caused by the layered phantom.

Using the fabricated TUT-arrays, we demonstrated a quad-mode US, PA, Doppler US, and optical fluorescence imaging capabilities. 6 MHz was selected as the imaging frequency as it was consistently one of the dual frequencies exhibited across all elements, despite different residual epoxy thicknesses (Figures 3, S1, S3). Three phantoms were used to validate the TUT-array for its capability of multimodal imaging. The schematic in Figure 6(a) represents a deep tissue phantom for validating US and PA imaging capabilities, Figure 6(b) schematic represents a blood flow phantom for Doppler US imaging, and Figure 6(c) demonstrates a fluorescence bead phantom used for showcasing optical fluorescence imaging through the TUT-array.

#### 2.3.1. USPA Imaging

The TUT-array was connected to the Vantage 256 ultrasound data acquisition system to perform real-time interleaved US and PA imaging on a tissue phantom prepared using a solution mixture of agarose and silica powder. The B-mode US imaging used a focused synthetic aperture beam transmitting strategy with a 16-element effective aperture at 0-degree beam steering. Each transmitted beam is focused at 15 mm away from the transducer surface. The US and PA imaging sequence is detailed in Materials and Methods: Imaging System and Data Acquisition Sequence. The phantom and imaging schematic is shown in Figure 6(a). The phantom consisted of four metal wire targets, each with a diameter of 50 *μ*m and dyed with India ink to generate strong photoacoustic contrast. Figure 6(a) shows the approximate positions of the 4 black wire targets along the imaging depth of the phantom, with approximately 5 mm distance between the targets. The tissue phantom also consisted of two ultrasound only targets (H1 and H2) in cylindrical shape filled with agar solution to mimic hypoechoic regions in the tissue medium (see Methods: Multimodal Imaging Phantom Preparation). The TUT-array was directly placed on top of the phantom and the laser light irradiated the phantom through the TUT-array, demonstrating the advantage of using the TUT-array for dual-modality USPA imaging with minimal coupling. To compare the US imaging performance of the TUT-array with a commercial ultrasound linear array, the same USPA phantom was also imaged with a linear probe (ATL L7-4, Philips) operated at the same 6 MHz frequency. The US image from the L7-4 is shown in Figure 6(d) and the USPA imaging results acquired by the TUT-array are demonstrated in Figures 6(e) and 6(f). Both the US images in Figures 6(d) and 6(e) clearly show the four micrometal wire targets (~5 mm in axial plane and ~3 mm in lateral plane) as these targets have different acoustic impedance compared to the background tissue-mimicking medium. The US image from the commercial probe L7-4 showed stronger contrasts for the wire targets and the background than that from the TUT-array with the same dynamic range. The wires were broadened on the lateral axis at deeper regions (>20 mm) in both the US images from L7-4 and TUT-array because the focus of the synthetic aperture beamforming was set to 15 mm depth. Simulation and experimental beam profile results at 0-degree steering angle in Figures 5(a) and 5(d) further confirmed that focal spot degrades at deeper depths. Considering the 50 *μ*m diameter metal wire as the point target, we calculated the full-width half maxima (FWHM) from the point spread function of target W1 (~15 mm deep in the phantom, as shown in Figures 6(d) and 6(e)) in the US images to quantify the lateral and axial resolutions of both the commercial probe and the TUT-array. The axial and lateral resolutions were found to be 307.6 *μ*m and 555.6 *μ*m, respectively, for L7-4 linear array and 894.2 *μ*m and 475.0 *μ*m, respectively, for the TUT-array. As expected, the TUT-array exhibits a comparable lateral resolution due to high focusing from the synthetic aperture transmit beam at 0-degree steering angle without grating lobes. However, due to limited bandwidth of the current TUT-array, the metal wire targets are not sharp in the depth axis as compared to the US image from L7-4. The ultrasound contrast from hypoechoic targets appeared as dark regions (red circled) in both the US images in Figures 6(d) and 6(e), with distinguishable boundary from the background ultrasound speckle contrast. The measured hypoechoic target diameter (~5 mm) is close to the real target size (4.75 mm). Due to better sensitivity of the commercial probe, the hypoechoic targets showed stronger contrasts than those in the TUT-array US image. The PA imaging result in Figure 6(f) showed depth-resolved optical absorption contrast from the four metal wires dyed with India ink. The locations of all four wires are clearly displayed at expected locations with sufficient PA contrast from the background. By measuring the FWHM at W1, the axial and lateral resolutions of the PA imaged wires are found to be 583.6 *μ*m and 363.1 *μ*m, respectively. The two hypoechoic targets are not observable in the PA image due to no significant light absorption from the transparent agar-only medium, as expected.

#### 2.3.2. Doppler Ultrasound

To demonstrate that the fabricated TUT-array is sensitive for mapping the microparticle motion-induced ultrasound frequency changes, Doppler ultrasound imaging was performed using a phantom consisted of a polyethylene tube running circulated blood mimicking fluid (BMF, particles of 5 *μ*m diameter). The schematic of this phantom is shown in Figure 6(b) and details are provided in Methods: Multimodal Imaging Phantom Preparation. A peristaltic pump was used to circulate the BMF in a loop through the tube (hence two parallel tubes in the field of view), while the TUT-array directly coupled to the phantom at an angle of 60° to the two tubes. The coregistered US and color Doppler image acquired and processed by Vantage 256 (see Methods: Imaging System and Data Acquisition Sequence). Figure 6(g) shows the same US speckle contrast from the two tubes in grayscale and an overlaid color Doppler image showed the opposite flow directions of the BMF in the two tubes in blue and red color scales. The measured size of these colored regions agreed well with the tube diameter of ~2 mm.

#### 2.3.3. Fluorescence Imaging

In the final step, we validated the feasibility of fluorescence imaging through the TUT-array. For this purpose, ultraviolet- (UV-) reactive fluorescence beads with 50 *μ*m beads diameter (Ultraglow, Techno Glow, Ennis, Texas, USA) were shaped to form “PSU” pattern within a rectangular area of 12 mm×3 mm. The TUT-array was then placed on top of the pattern. Under the 120 Watts UV light excitation, the captured fluorescence emission signal from the “PSU” could be easily distinguished from the background in Figure 6(c). The discontinuities in the fluorescence image are due to the translucent kerfs in the TUT-array.

## 3. Discussions

In this paper, a transparent lithium niobate-based TUT-array was fabricated and validated for multimodal optical and ultrasound imaging applications. It is, to the best of our knowledge, the first transparent ultrasound linear array using bulk piezoelectric material. We successfully demonstrated the feasibility of the TUT-array for a quad-mode US, PA, Doppler US, and fluorescence imaging in real-time. Dual-modality USPA imaging results using the TUT-array demonstrated the potential of the TUT-array to acquire both US (30 frames per second) and PA (10 frames per second, limited by laser firing rate) images with a bare minimum acoustic coupling between the array and the imaging subject. The ability to illuminate light through the TUT-array and into the imaging object without any additional optical components not only reduced the complexity for building a multimodal ultrasound and optical imaging platform but also helped eliminate the shadow illumination problems commonly observed in the conventional B-mode dual-modality USPA imaging systems. Further, the experiments on blood flow mimicking phantoms demonstrated that the TUT-array is also capable of mapping the direction of blood flow using color Doppler ultrasound. In addition, the high optical transparency of the TUT-arrays was exploited for imaging fluorescence objects. Together, these experiments demonstrated the feasibility of developing multimodality optical and ultrasound imaging platform based on the TUT-array technology, in particular, the space constrained miniaturized multimodal endoscopy devices. For example, the proposed array may be made in an endoscopy form to integrate the current standard optical and ultrasound endoscopy [65–67] into one platform for image-guided biopsy for early cancer detection. Using this platform, the patient would only undergo one endoscopy procedure, but providing rich multimodal information: US-based structural information, Doppler US-based blood flow information, PA-based blood oxygenation information, and fluorescence-enhanced tumor metabolism (e.g., NADH). Such a comprehensive information is needed to assess the tissue function and pre and posttreatment efficiency [68].

However, the current TUT-array needs further optimization on below mentioned challenges before it becomes clinically applicable. Backing layer bonding: The uneven bonding epoxy thickness between the conductive glass slide and the piezoelectric material for ground connection led to variations in pulse-echo responses, resulting in three major frequency responses observed across the 64 array elements. Our simulations confirmed that epoxy thickness between the LN and the conductive glass changes the electrical impedance and acoustic response of the element. One of the ways to overcome this issue in future is to replace conductive glass slide with a transparent electrode-coated epoxy block to serve as a homogeneous backing layer. Sensitivity and bandwidth: Comparing the ultrasound imaging capabilities of the current TUT-array to a commercial linear probe demonstrated that the TUT-array imaged the phantom targets with much lower contrast and lower axial resolution due to lower sensitivity and deteriorated bandwidths. In the future, the TUT-array sensitivity can be improved by optimizing each acoustic stacking layer in the TUT fabrication. First of all, a transparent piezoelectric material with higher piezoelectricity can be employed, such as the alternative current (AC) poled lead magnesium niobate-lead titanate (PMN-PT), which exhibited a $d_{33}$ of 2200 pC/N as compared to LN with a $d_{33}$ of 350 pC/N [59, 69]. The bandwidth and sensitivity are also reduced by the glass backing layer. The glass bonded to the back side of the linear array induced the mass-loading effect and this resulted in a dual-frequency nature. Additionally, the conductive glass slide is not an ideal backing material to the ultrasound transducer because its acoustic impedance (~11 MRayls) is not matched to that of LN piezoelectric material (~34 MRayls) and the glass slide is not a good acoustic absorbing or damping material. These factors contributed to the significant ringing in the detected ultrasound pulse-echo waveform, and therefore reducing the bandwidth and deteriorating the axial resolution. The above-mentioned conductive epoxy block as backing may also serve to reduce the significant mass-loading effect. Additionally, a novel translucent matching layer is also needed to improve the sensitivity and bandwidth of the transducer, such as our recently reported matching layer that uses translucent glass beads [70]. Dicing method: In order to maintain a common ground as an electrode, a subdicing method was employed in the current TUT-array fabrication. However, as a result, no beam steering and focusing capabilities could be exploited for ultrasound image formation due to concerns for strong grating and side lobes when the beam was steered at an angle (Figures 5(e) and 5(f)). This subdicing method limited the ultrasound transmit beamforming to be at 0 degrees, and therefore, a synthetic aperture beamforming method was employed to generate B-mode ultrasound images. In the future, a fully diced TUT-array geometry will not only help reduce the crosstalk but also allow beam steering capabilities. System electrical resistance: Lastly, the current TUT-array also suffered from high resistance that mainly contributed by the transparent electrode-ITO. The high resistivity from ITO may increase loss and make it challenging to electrical impedance match with the driving circuit. Strategies to improve the system conductivity such as Cr/Au or Cr/Cu coating around the ITO [71] or new transparent electrodes with lower resistivity such as strontium niobate (SrNbO_3_) [72] will be investigated in the future to improve the transducer performance.

Despite these limitations, the new TUT-array fabricated using transparent LN demonstrated potential advantages in realizing an integrated multimodal optical, US and PA imaging device for providing complementary structural, functional, and molecular contrasts and spatial resolutions. The TUT platform can be scaled to develop multimodal devices of different length scales such as miniaturized endoscopy or wearable devices and therefore may open new avenues for combined optical, ultrasound, and photoacoustic imaging in preclinical and clinical studies.

## 4. Materials and Methods

### 4.1. TUT-Array Fabrication and Packaging

**Figure 7:**
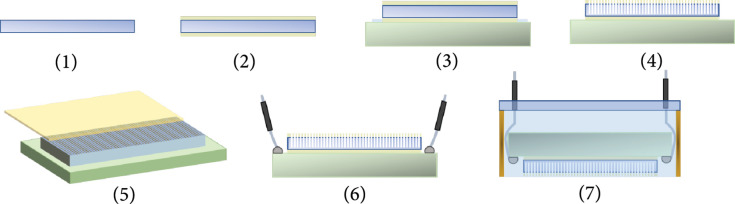
Step-wise fabrication process of the TUT-array. Step 1: a double-side polished lithium niobate. Step 2: indium tin oxide was deposited on two sides of the lithium niobate as conductive electrode. Step 3: the coated lithium niobate was bonded to a conductive glass slide. Step 4: the elements were created by dicing the lithium niobate to 80% depth. Step 5: the custom-made flexible cable was bonded to the array by anisotropic conductive film bonding. Step 6: the wires were connected to the conductive glass slide as ground. Step 7: the array was then placed inside a brass housing and filled with transparent epoxy.

The TUT-array was fabricated by dicing a rectangular double-side polished transparent lithium niobate (LN) piece. The step-by-step fabrication process is illustrated in Figure 7(a). Step (1): a 0.5 mm thick 36° Y-cut LN wafer (Precision Micro Optics, Burlington, MA, USA) was used for the designed center frequency of 6.5 MHz. Step (2): 200 nm ITO was deposited on both sides of the LN as a transparent and conductive electrode. Step (3): to form a common ground electrode, a one side ITO-coated glass slide was hard pressed to the ITO-coated LN using a small drop of transparent epoxy (EPO-TEK 301, Epoxy Technologies Inc., Billerica, MA, USA) as the bonding agent. The custom-made press-bonding platform was lapped and polished to ensure a flat surface during the bonding process. Care was taken to allow the bonding strength was enough to squeeze out the epoxy but not damaging the LN or the conductive glass slide. Step (4): a high-precision dicing machine (K&S 982-6, Giorgio Technology sales/service, Mesa, AZ, USA) with a 70 *μ*m thick blade was used to dice out 64 elements on the LN substrate with 0.3 mm pitch. The dicing depth was kept to be ~80% of the depth (400 *μ*m), so the ground electrode was intact. Step (5): we custom designed and fabricated a 50 *μ*m thick polyimide base flexible cable with 3 *μ*m thick and 100 *μ*m wide copper traces (70 channels with 0.3 mm pitch at the array end and 0.5 mm pitch at the other end connected to Vantage 256). The flexible cable was bonded to the TUT-array by anisotropic conductive film (ACF) bonding. The ACF bonding procedure is similar to previously reported article [73]. In brief, an 18 *μ*m thick and 1.5 mm wide ACF tape (AC-7206 U ACF, Hitachi, Tokyo, Japan) was tacked on the cable with 1 MPa pressure and 90°C for 10 seconds and then aligned to the transparent array elements. To maximize the transparent aperture and minimize the acoustic mismatching effect on array elements, the polyimide flex cable was bonded to the elements with minimal overlap (~2 mm). Next, the TUT-array/ACF tape/flex cable assembly was applied with 1 MPa pressure at 180°C for 25 seconds to allow trapping of conductive particles in the conductors. Step (6): ground wires were connected to the ITO-coated glass plate electrode with help of a small blob of silver epoxy (E-solder 3022, Von Roll Isola Inc., New Haven, CT, USA). Step (7): for protection and electromagnetic shielding purpose, a rectangular brass housing (size 12.7 mm×38.1 mm×10 mm) was used to surround the device and connected with the ground electrode. A transparent epoxy (EPO-TEK 301, Epoxy Technologies Inc., Billerica, MA, USA) was then poured inside the brass housing to fill the kerfs and serve as the second backing layer. A glass slide was placed on top of the brass tube to form a leveled epoxy layer. Step (8): lastly, an 80 *μ*m thick Parylene C film was deposited on the full device to serve as the matching layer and waterproof layer. Matching layer can improve the acoustic wave transmission efficiency, and moreover, waterproof layer is critical to this device as ACF bonding tape is susceptible to humidity and can easily detach from the bonding. The fabricated linear array was then connected to a 70-pin 0.5 mm pitch commercial interface board (FPC050P070, Chip Quik Inc., Ancaster, ON, USA) by ACF bonding the interface end of the custom-designed flexible cable (same process as described above). Copper tape wrapped 42 AWG coaxial cables (2420/42 WH-100, Daburn Electronics & Cable, Dover, NJ, USA) were drawn from the interface board to Vantage 256, a MATLAB-based ultrasound data acquisition and image reconstruction platform, to perform the array characterization and US, PA, and Doppler US imaging validations. The final packaged linear array is schematically depicted in Supplementary Figure S4.

### 4.2. Beam Profile Hydrophone Measurement

The TUT-array was connected to Vantage 256, transmit profiles were modified to fire 16 elements centered around element #32 (i.e., elements #25, #26, …, #39, and #40) for three different angles: 0 degree, -10 degree, and 10 degree. A calibrated hydrophone (HGL0085, Onda, Sunnyvale, CA, USA) was then raster scanned the beam profile using a two-axis linear stage (NRT-1000, Thorlabs Inc., Newton, NJ, USA). The acquired hydrophone signal was then averaged 16 times, amplified by a preamplifier with a 20 dB gain, and then captured by a high-speed data acquisition system (Razormax 16, GaGe, Lockport, IL, USA). The resultant beam profile was then normalized to its highest pressure and plotted in dB scale.

### 4.3. Imaging System and Data Acquisition Sequence

#### 4.3.1. USPA Imaging

A portable optical parametric oscillator (OPO) laser source (Phocus Mobile, Opotek Inc., Carlsbad, CA, USA) was integrated with the Vantage system to provide 10 Hz, pulsed (5-7 ns pulses with peak output pulse energy of 120 mJ at 730 nm) and tunable (690-970 nm) optical illumination needed for multispectral PA imaging. A custom designed optical fiber bundle (FiberOptics Inc., CA, USA) that has a similar aperture area as the TUT-array aperture was employed to couple the light from the laser source to the array. Because the subdiced TUT linear array introduces strong grating lobes in steering angles, a focused synthetic aperture beam transmitting strategy was employed with 0-degree steering angle. Each B-mode US frame involved 64 focused transmit and receive acquisitions with 8 transmitters active on each side of the transmitting element (i.e., effective transmitting aperture is 16 element where possible, similar to Materials and Methods: Beam Profile Hydrophone Measurement). All 64 elements are active for US data acquisition. Each effective aperture emits a 0-degree beam that is focused at 15 mm depth from the transducer surface, similar to the plot shown in Figure 5(d). Time allotted for each focused beam acquisition was 150 *μ*s, leading to a total B-mode frame acquisition time of 9.6 ms. The data corresponding to this frame is then transferred to the host for reconstruction and display. After every 3 US frame acquisitions, the control sequence waits for the laser trigger which happens every 100 ms as we have used a 10 Hz pulse repetition frequency laser. One PA frame is acquired at the laser trigger event with all 64 elements active in the receive mode, and the received PA data is transferred to the host for reconstruction and display of (1) the PA, (2) latest US frame in buffer, and (3) coregistered image of latest US and PA frames. Overall US frame rate achieved was 30 frames per second (FPS) and the PA imaging frame rate is 10 FPS, limited by the laser pulse repetition frequency of the laser. A function generator was used in master mode to synchronize both Vantage and the laser system, by setting the required time delays and thus allowing a proper interleaved, coregistered US + PA image formation. The corresponding timing diagram is demonstrated in Supplementary Figure S5.

#### 4.3.2. Doppler Ultrasound Imaging

For acquiring the Doppler ensemble, 14 plane wave pulses were transmitted at a steering angle of 12° and at a pulse repetition rate of 3 kHz. The transmit pulses used for Doppler ultrasound acquisition consisted of three complete cycles in contrast to one cycle used for B-mode acquisition to get higher Doppler sensitivity. All 64 transmit and receive channels were active for each plane wave acquisition of the Doppler ensemble. The velocity and power Doppler processing was asynchronous with respect to the Doppler ensemble acquisition and was performed using the Doppler processing routines provided by the Verasonics platform.

### 4.4. Multimodal Imaging Phantom Preparation

#### 4.4.1. USPA Imaging Phantom

Four 50 *μ*m diameter micrometal wires (W1-W4) were dyed using India ink to generate both ultrasound and photoacoustic contrasts. Micrometal wires were placed 5 mm apart from each other on the axial plane and 3 mm apart from each other on the lateral plane in an acrylic tank filled with a solution mixture of agar and silica beads. Silica beads and agar are mixed with water at 1% and 1.5% weight ratio, respectively. Here, the silica beads were used to mimic the background ultrasound speckle contrast. Then, 1% agar solution was filled inside the 4.75 mm diameter cylindrical columns, next to the pencil leads, to serve as hypoechoic targets inside the tissue phantom for US imaging validation.

#### 4.4.2. Blood Flow Doppler Phantom

A blood-mimicking fluid (BMF-US, Shelley Automation, nylon particles with 5 *μ*m diameter, 1548±5 m/s speed of sound, $1037\pm 2\;\text{kg}/{\text{m}}^{3}$ fluid density, and 1.82% concentration) is circulated inside a polyethylene tube (outer diameter: 2.08 mm, inner diameter: 1.57 mm) using a peristaltic pump (model 3386, Cole-Parmer, Vernon Hills, IL, USA). The tube was partially submerged inside a tank filled with 1.5% agar solution. The tube was placed at 60 degrees to the imaging plane of the TUT-array as shown in Figure 6(b).

## Data Availability

Data underlying the results presented in this paper are not publicly available at this time but may be obtained from the authors upon reasonable request.

## Supplementary Materials

Supplementary Materials Figure S1: comparison of experimental pulse-echo waveforms of typical elements of the array with simulated pulse-echo waveforms of TUT-array element with different residual epoxy thickness. Figure S2: combined acoustic and electrical crosstalk measurement at frequencies between 3 MHz and 11 MHz for element #4. Figure S3: comparison of experimental electrical impedance results of typical elements of the array with simulated impedance analysis results of TUT-array array element with different residual epoxy thickness. Figure S4: schematic of the TUT-array connection to the Vantage 256 ultrasound data acquisition system. Figure S5: timing diagram of the US and PA imaging sequence.
