# Supplementary material for: A Transparent Ultrasound Array for Real-Time Optical, Ultrasound, and Photoacoustic Imaging
Source: BME Front. 2022 Jun 8;2022:9871098. doi: 10.34133/2022/9871098 (PMC10521654; doi:10.34133/2022/9871098)
Supplement: Supplementary Materials — Figure S1: comparison of experimental pulse-echo waveforms of typical elements of the array with simulated pulse-echo waveforms of TUT-array element with different residual epoxy thickness. Figure S2: combined acoustic and electrical crosstalk measurement at frequencies between 3 MHz and 11 MHz for element #4. Figure S3: comparison of experimental electrical impedance results of typical elements of the array with simulated impedance analysis results of TUT-array array element with different residual epoxy thickness. Figure S4: schematic of the TUT-array connection to the Vantage 256 ultrasound data acquisition system. Figure S5: timing diagram of the US and PA imaging sequence. [file 9871098.f1.zip › Supplementary Material Round 2_Submission.pdf]

A Transparent Ultrasound Array for Real-time Optical, Ultrasound and Photoacoustic Imaging

Haoyang Chen[1], Sumit Agrawal[1], Mohamed Osman[1], Josiah Minotto[1], Shubham Mirg[1], Jinyun Liu[1], Ajay Dangi[1], Quyen Tran[2], Thomas Jackson[2], and Sri-Rajasekhar Kothapalli[1,3,4*]

[1]Department of Biomedical Engineering, The Pennsylvania State University, University Park, PA 16802, USA

[2]School of Electrical Engineering and Computer Science, The Pennsylvania State University, University Park, PA 16802, USA

[3]Penn State Cancer Institute, The Pennsylvania State University, Hershey, PA 17033, USA

[4]Graduate Program in Acoustics, The Pennsylvania State University, University Park, PA 16802, USA

*Corresponding author. Email: srkothapalli@psu.edu

Supplementary Figures

- **Page 1: Figure S1. Comparison of experimental pulse-echo wave forms of typical elements of the array with simulated pulse-echo waveforms of TUT-array element with different residual epoxy thickness.**

- **Page 2: Figure S2. Combined acoustic and electrical crosstalk measurement at frequencies between 3 MHz and 11 MHz for element #4.**

- **Page 3: Figure S3. Comparison of experimental electrical impedance results of typical elements of the array with simulated impedance analysis results of TUT-array element with different residual epoxy thickness.**

- **Page 4: Figure S4. Schematic of the TUT-array connection to the Vantage 256 ultrasound data acquisition system.**

- **Page 5: Figure S5. Timing diagram of the US and PA imaging sequence.**

Supplementary Figures

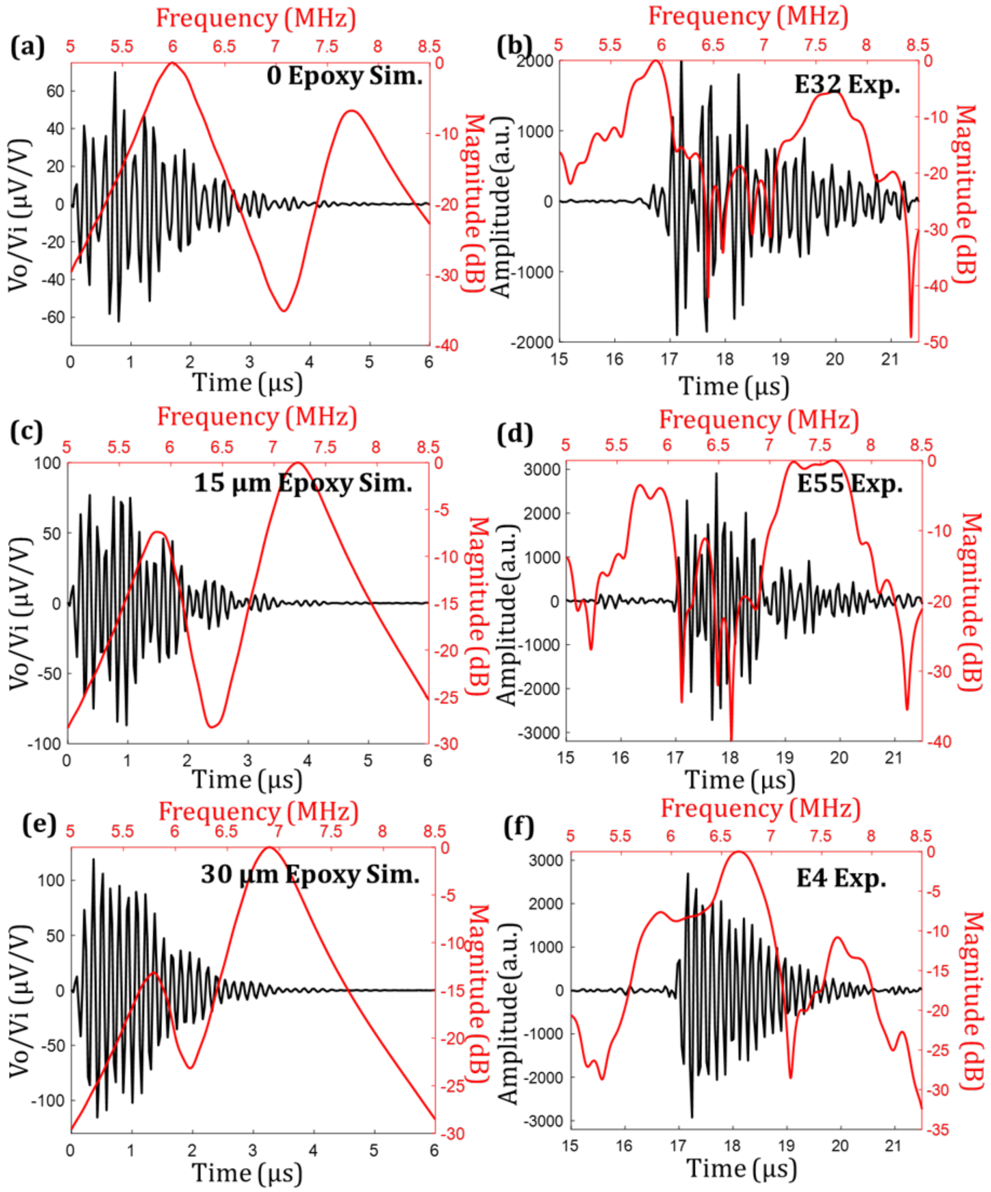

Figure S1. Comparison of experimental pulse-echo wave forms of typical elements of the array with simulated pulse-echo waveforms of TUT-array element with different residual epoxy thickness. (a) PiezoCAD simulated pulse-echo waveform of TUT element with 0 μm residual epoxy thickness matches with **(b)** experimental pulse-echo response of element 32 (E32). **(c)** PiezoCAD simulated pulse-echo waveform of TUT element with 15 μm residual epoxy thickness matches with **(d)** experimental pulse-echo response of element 55 (E55) **(e)** PiezoCAD simulated pulse-echo waveform of TUT element with 30 μm residual epoxy thickness matches with **(f)** experimental pulse-echo response of element 4 (E4). Black line represents the time domain signal and the red line represents the corresponding frequency response.

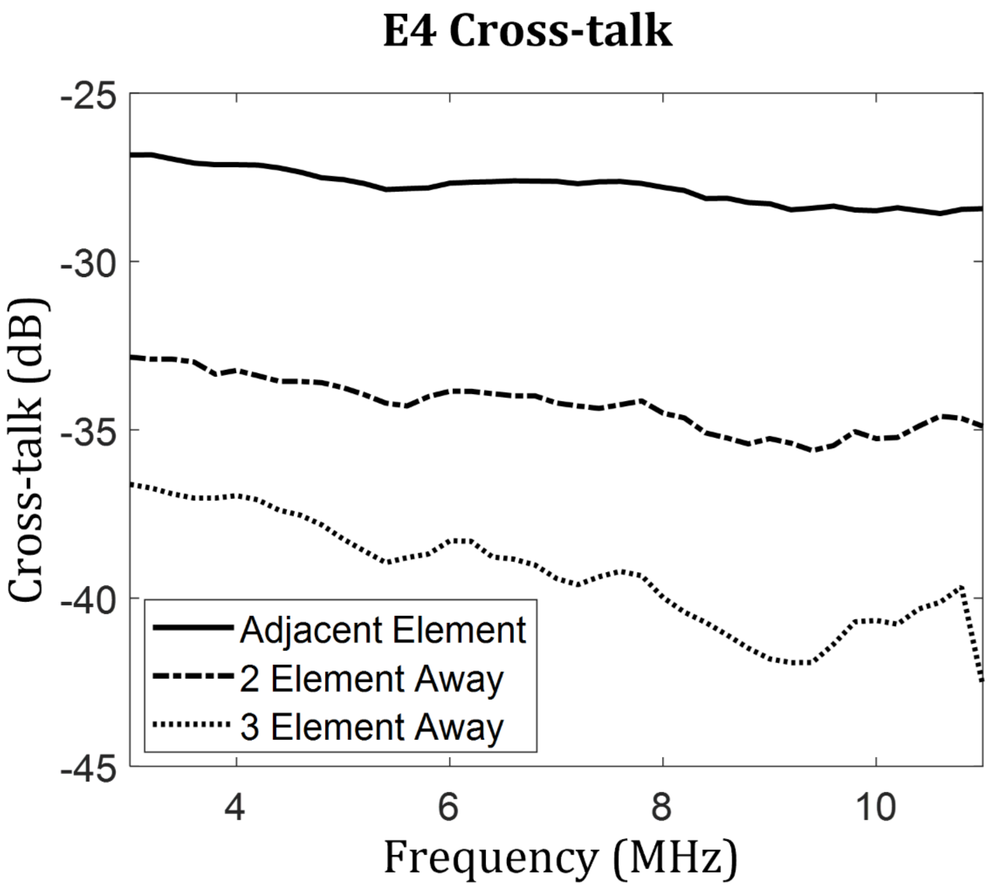

Figure S2. Combined acoustic and electrical crosstalk measurement at frequencies between 3 MHz and 11 MHz for element #4.

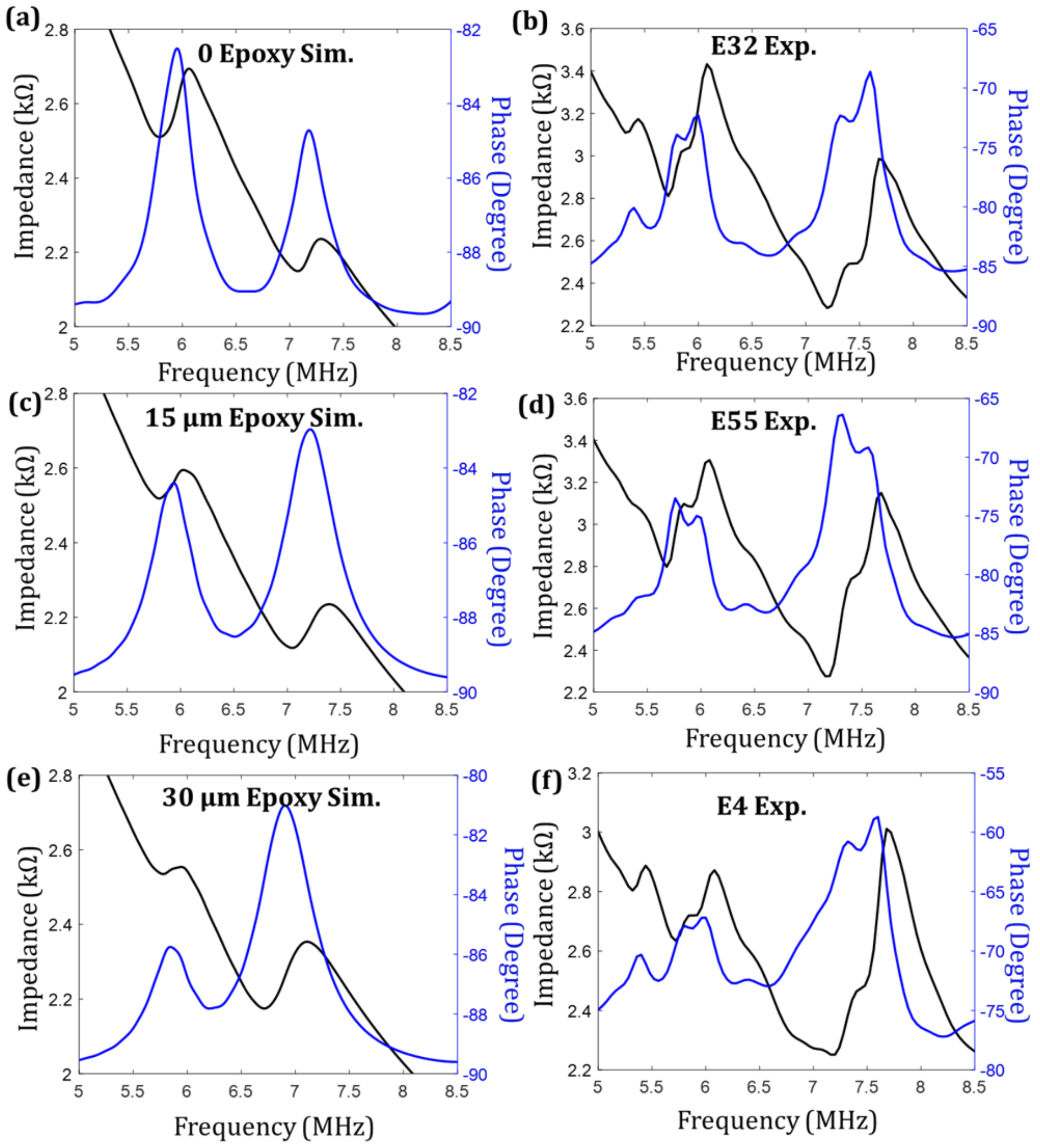

Figure S3. Comparison of experimental electrical impedance results of typical elements of the array with simulated impedance analysis results of TUT-array element with different residual epoxy thickness. (a) PiezoCAD simulated input electrical impedance waveform with 0 μm residual epoxy thickness and corresponding **(b)** experimental impedance analysis result from element 32 (E32) **(c)** PiezoCAD simulated input electrical impedance waveform of TUT element with 15 μm residual epoxy thickness and corresponding **(d)** experimental impedance analysis result from element 55 (E55). **(e)** PiezoCAD simulated input electrical impedance waveform with 30 μm residual epoxy thickness and corresponding **(f)** experimental impedance analysis result from element 4 (E4). Black line represents the impedance curve and the blue line represents the phase curve.

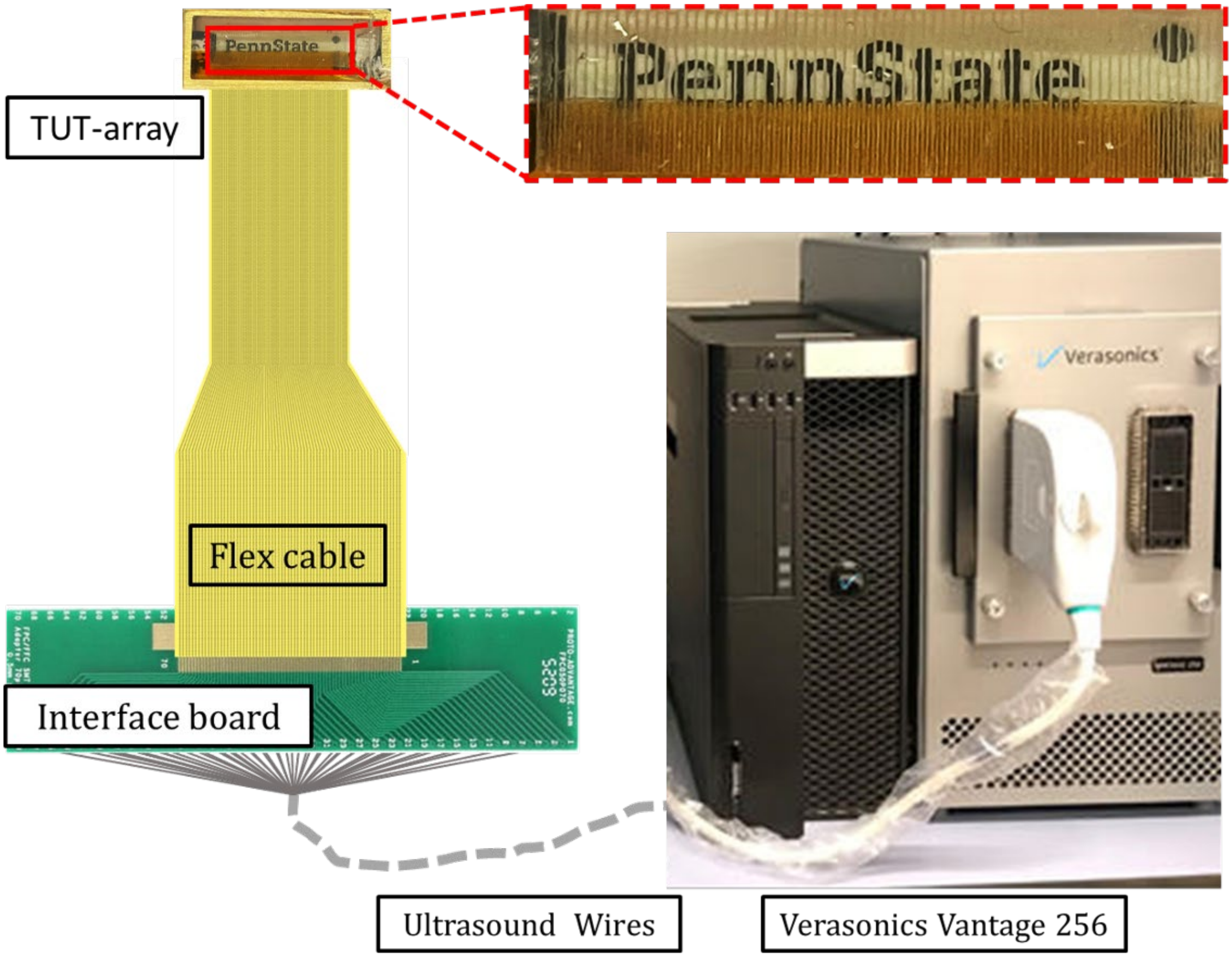

Figure S4. Schematic of the TUT-array connection to the Vantage 256 ultrasound data acquisition system. Flex cable has 70 connectors with 0.3 mm pitch on one side bonded to the TUT-array, while the other side with 0.5 mm pitch bonded with an interface board. The interface board connects with the Verasonics Vantage 256 through open-wires from a commercial linear probe.

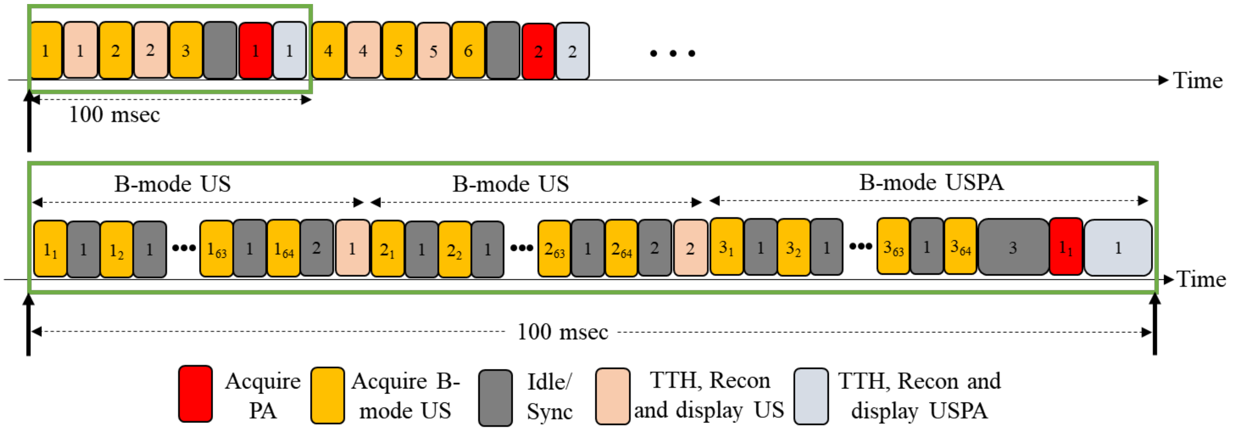

Figure S5. Timing diagram of the US and PA imaging sequence. To form one US image, synthetic aperture with an effective aperture size of 16-element is focused at 15 mm depth away from the transducer surface for 64 focused beam transmitting profiles with an idle time of 150 μs (idle time 1). After 64 transmit profile, idle time of 7 ms (idle time 2) was then allocated for transferring the ultrasound data to the host, and US imaging reconstruction and display. For the last US frame in every 100 ms, the US data is transferred after receiving an external trigger from the 10 Hz laser, then both the US and PA image data were transferred to the host and display the US, PA, and co-registered US and PA images. TTH: transfer to host.
