# Supplementary figures and images for: A Transparent Ultrasound Array for Real-Time Optical, Ultrasound, and Photoacoustic Imaging
Source: BME Front. 2022 Jun 8;2022:9871098. doi: 10.34133/2022/9871098 (PMC10521654; doi:10.34133/2022/9871098)

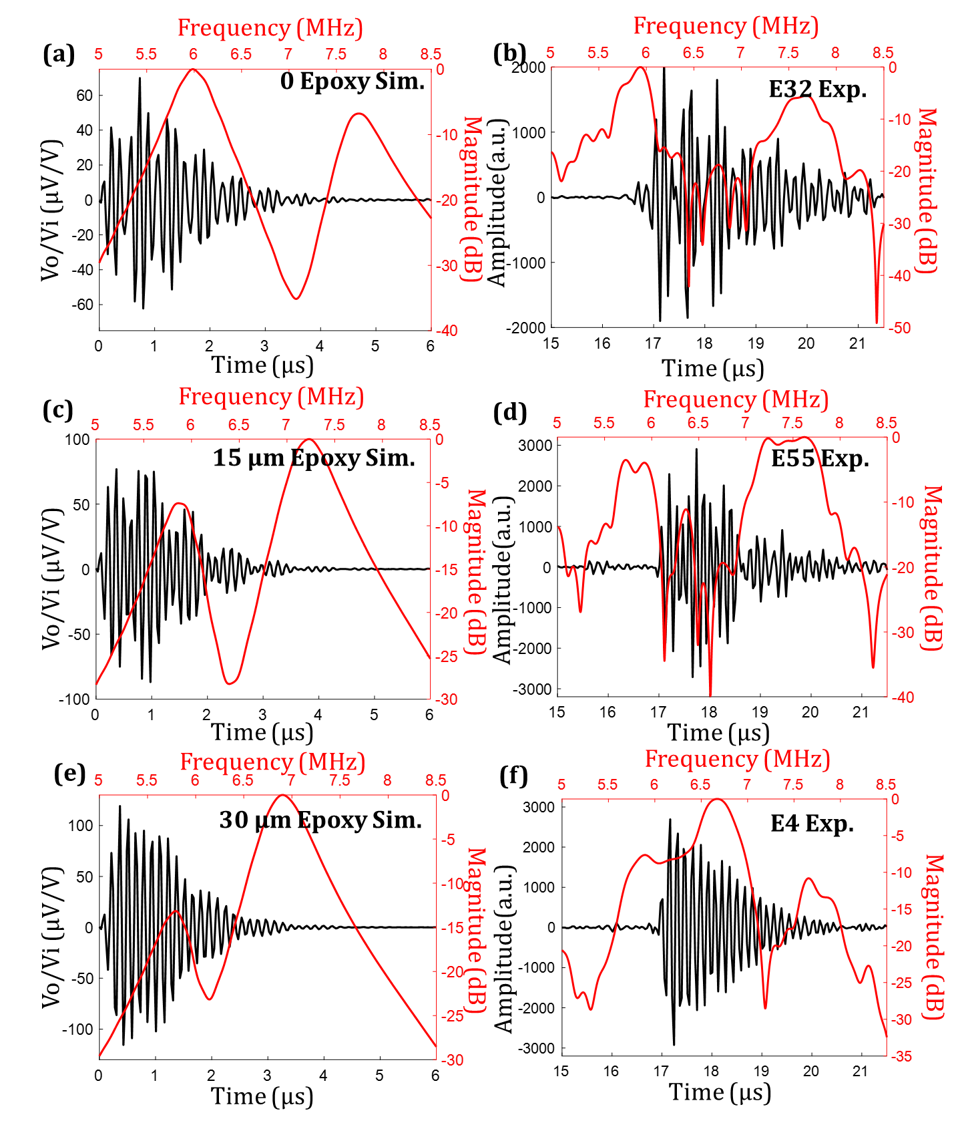

Supplement: Supplementary Materials — Figure S1: comparison of experimental pulse-echo waveforms of typical elements of the array with simulated pulse-echo waveforms of TUT-array element with different residual epoxy thickness. Figure S2: combined acoustic and electrical crosstalk measurement at frequencies between 3 MHz and 11 MHz for element #4. Figure S3: comparison of experimental electrical impedance results of typical elements of the array with simulated impedance analysis results of TUT-array array element with different residual epoxy thickness. Figure S4: schematic of the TUT-array connection to the Vantage 256 ultrasound data acquisition system. Figure S5: timing diagram of the US and PA imaging sequence. [file 9871098.f1.zip › R2_FigS1_v2.png]

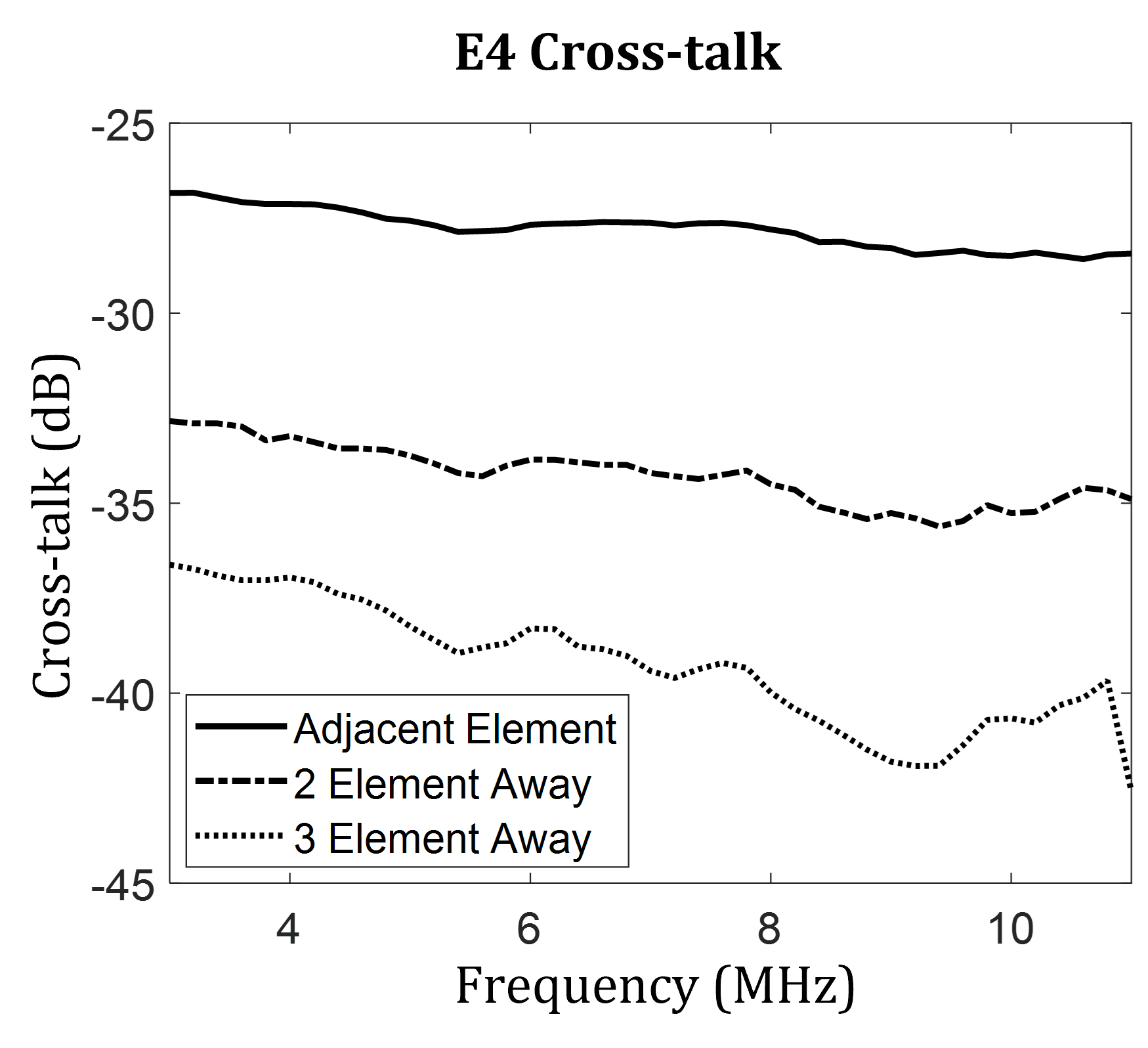

Supplement: Supplementary Materials — Figure S1: comparison of experimental pulse-echo waveforms of typical elements of the array with simulated pulse-echo waveforms of TUT-array element with different residual epoxy thickness. Figure S2: combined acoustic and electrical crosstalk measurement at frequencies between 3 MHz and 11 MHz for element #4. Figure S3: comparison of experimental electrical impedance results of typical elements of the array with simulated impedance analysis results of TUT-array array element with different residual epoxy thickness. Figure S4: schematic of the TUT-array connection to the Vantage 256 ultrasound data acquisition system. Figure S5: timing diagram of the US and PA imaging sequence. [file 9871098.f1.zip › R2_FigS2_v1.png]

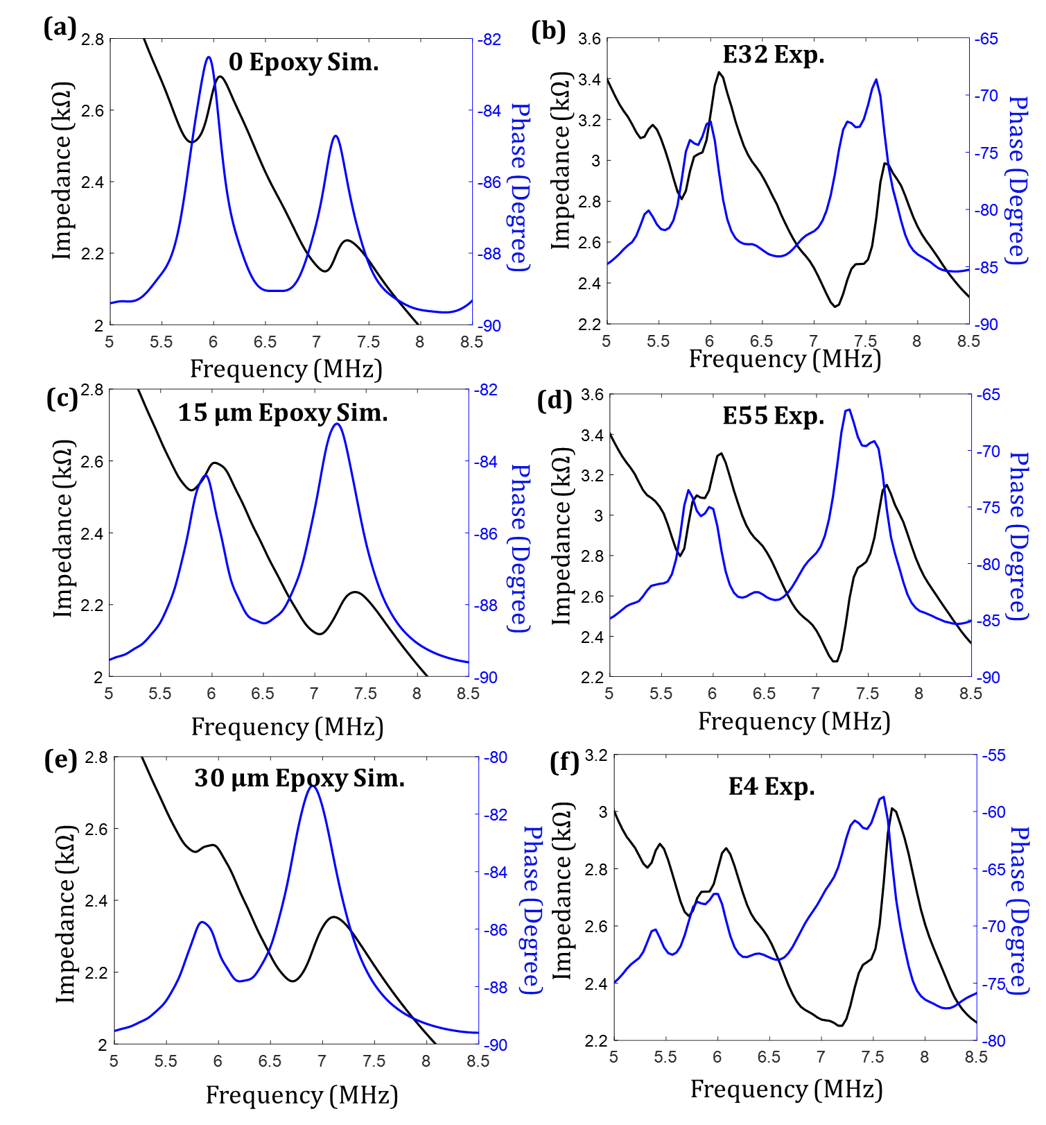

Supplement: Supplementary Materials — Figure S1: comparison of experimental pulse-echo waveforms of typical elements of the array with simulated pulse-echo waveforms of TUT-array element with different residual epoxy thickness. Figure S2: combined acoustic and electrical crosstalk measurement at frequencies between 3 MHz and 11 MHz for element #4. Figure S3: comparison of experimental electrical impedance results of typical elements of the array with simulated impedance analysis results of TUT-array array element with different residual epoxy thickness. Figure S4: schematic of the TUT-array connection to the Vantage 256 ultrasound data acquisition system. Figure S5: timing diagram of the US and PA imaging sequence. [file 9871098.f1.zip › R2_FigS3_v2.png]

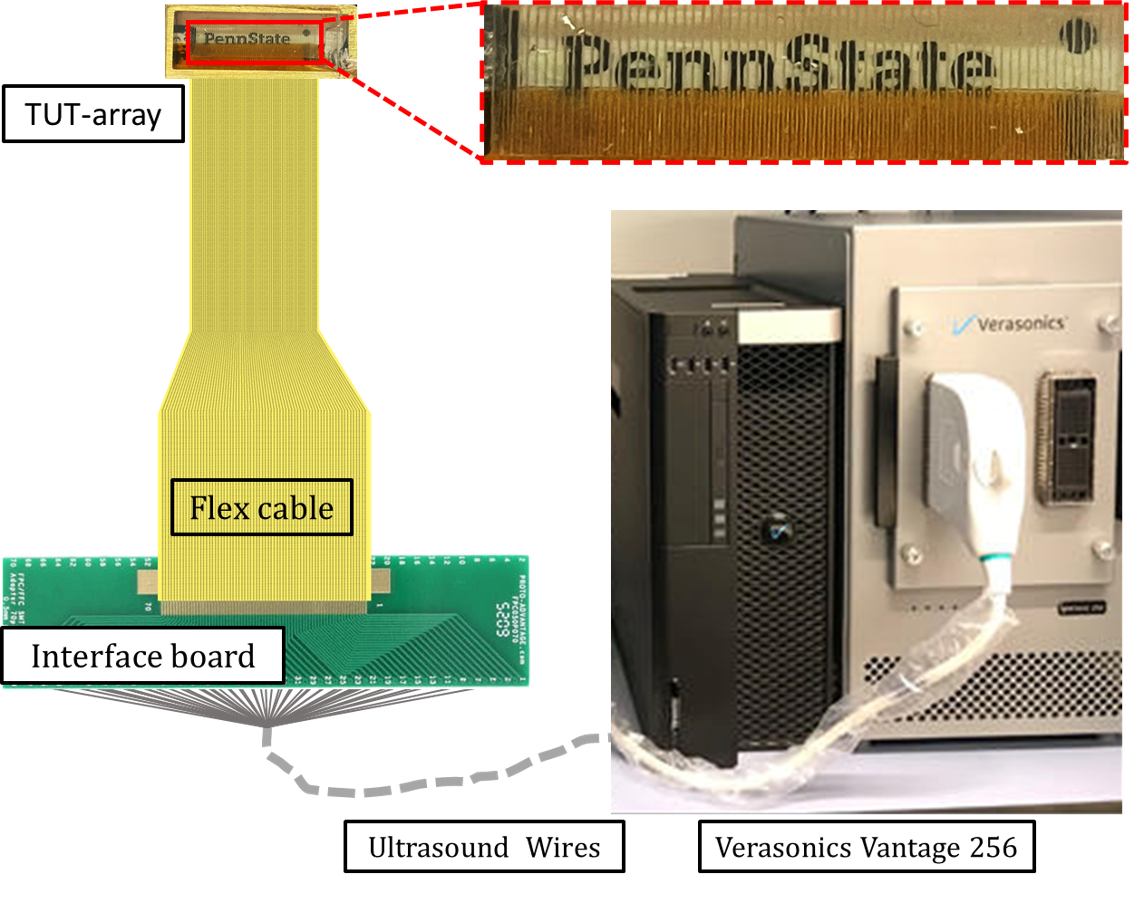

Supplement: Supplementary Materials — Figure S1: comparison of experimental pulse-echo waveforms of typical elements of the array with simulated pulse-echo waveforms of TUT-array element with different residual epoxy thickness. Figure S2: combined acoustic and electrical crosstalk measurement at frequencies between 3 MHz and 11 MHz for element #4. Figure S3: comparison of experimental electrical impedance results of typical elements of the array with simulated impedance analysis results of TUT-array array element with different residual epoxy thickness. Figure S4: schematic of the TUT-array connection to the Vantage 256 ultrasound data acquisition system. Figure S5: timing diagram of the US and PA imaging sequence. [file 9871098.f1.zip › R2_FigS4_v1.png]

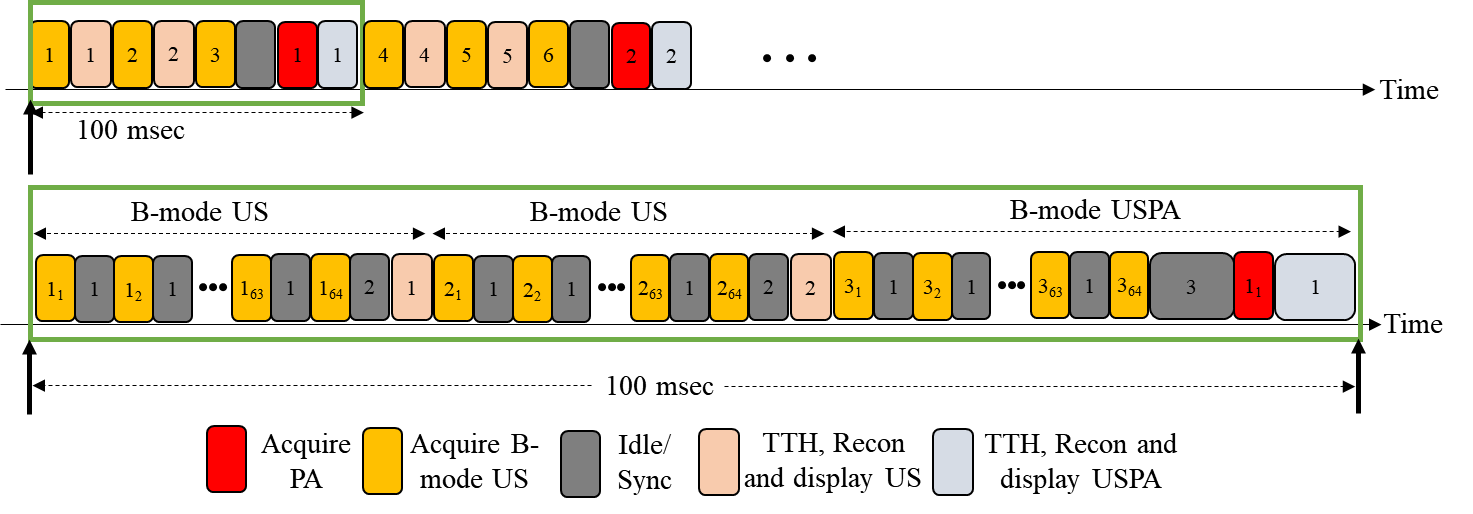

Supplement: Supplementary Materials — Figure S1: comparison of experimental pulse-echo waveforms of typical elements of the array with simulated pulse-echo waveforms of TUT-array element with different residual epoxy thickness. Figure S2: combined acoustic and electrical crosstalk measurement at frequencies between 3 MHz and 11 MHz for element #4. Figure S3: comparison of experimental electrical impedance results of typical elements of the array with simulated impedance analysis results of TUT-array array element with different residual epoxy thickness. Figure S4: schematic of the TUT-array connection to the Vantage 256 ultrasound data acquisition system. Figure S5: timing diagram of the US and PA imaging sequence. [file 9871098.f1.zip › R2_FigS5_v1.png]
